# Nuclear Peroxisome Proliferator-Activated Receptors (PPARs) as Therapeutic Targets of Resveratrol for Autism Spectrum Disorder

**DOI:** 10.3390/ijms20081878

**Published:** 2019-04-16

**Authors:** Rita Barone, Renata Rizzo, Giovanni Tabbì, Michele Malaguarnera, Richard E. Frye, Jean Bastin

**Affiliations:** 1 Child Neurology and Psychiatry Unit, Department of Clinical and Experimental Medicine, University of Catania, 95123 Catania, Italy; rerizzo@unict.it; 2 Referral Centre for Inborn Metabolic Diseases, Department of Clinical and Experimental Medicine, University of Catania, 95123 Catania, Italy; 3 Institute for Polymers, Composites and Biomaterials (IPCB), National Research Council (CNR), 95126 Catania, Italy; 4 Institute of Crystallography (IC), National Research Council (CNR), 95126 Catania, Italy; giovanni.tabbi@cnr.it; 5 Laboratory of Neurobiology, Centro Investigación Príncipe Felipe, 46012 Valencia, Spain; michele.malaguarnera@gmail.com; 6 Research Center “The Great Senescence”, University of Catania, 95123 Catania, Italy; 7 Barrow Neurologic Institute at Phoenix Children’s Hospital, Phoenix, AZ 85016, USA; rfrye@phoenixchildrens.com; 8 Department of Child Health, University of Arizona College of Medicine, Phoenix, AZ 85016, USA; 9 Centre de Recherche des Cordeliers, 75006 Paris, France; jean.bastin@inserm.fr; 10 Sorbonne Université, USPC, 75006 Paris, France; 11 Université Paris Descartes, 75006 Paris, France; 12 Université Paris Diderot, 75006 Paris, France; 13 INSERM UMR-S 1138, 75006 Paris, France

**Keywords:** autism spectrum disorders, mitochondrial fatty acid β-oxidation metabolism, PPARs agonists, resveratrol

## Abstract

Autism spectrum disorder (ASD) is a neurodevelopmental disorder characterized by defective social communication and interaction and restricted, repetitive behavior with a complex, multifactorial etiology. Despite an increasing worldwide prevalence of ASD, there is currently no pharmacological cure to treat core symptoms of ASD. Clinical evidence and molecular data support the role of impaired mitochondrial fatty acid oxidation (FAO) in ASD. The recognition of defects in energy metabolism in ASD may be important for better understanding ASD and developing therapeutic intervention. The nuclear peroxisome proliferator-activated receptors (PPAR) α, δ, and γ are ligand-activated receptors with distinct physiological functions in regulating lipid and glucose metabolism, as well as inflammatory response. PPAR activation allows a coordinated up-regulation of numerous FAO enzymes, resulting in significant PPAR-driven increases in mitochondrial FAO flux. Resveratrol (RSV) is a polyphenolic compound which exhibits metabolic, antioxidant, and anti-inflammatory properties, pointing to possible applications in ASD therapeutics. In this study, we review the evidence for the existing links between ASD and impaired mitochondrial FAO and review the potential implications for regulation of mitochondrial FAO in ASD by PPAR activators, including RSV.

## 1. Introduction

Autism spectrum disorder (ASD) is a lifelong condition characterized by persistent deficits in social communication and social interaction across multiple contexts and restricted and/or repetitive patterns of behavior and/or activities that must be present in the early developmental period [1]. ASD affect males more than females by a ratio of 4:1. The current prevalence is estimated to be one in 59 children aged 8 years of age in the US [2]. The precise etiology remains unknown although various factors are involved including genetic factors, environmental toxins and stressors, impaired immune responses, mitochondrial dysfunction, and neuroinflammation [3].

Classic inborn errors of metabolism (IEM) are monogenic diseases that affect a subgroup of ASD patients, accounting for 1–3% of children with ASD [4,5,6]. Among genetic metabolic diseases, primary mitochondrial diseases (MD) affect the structure or function of the mitochondria, because of either nuclear DNA (nDNA), or mitochondrial DNA (mtDNA) mutations [7]. MD are heterogeneous disorders with complex, poorly understood pathogenesis. Very diverse phenotypes are encountered in MD, possibly affecting numerous organs, and frequently involving the central nervous system (CNS). Mitochondrial bioenergetic defects have been associated with ASD because the brain is one of the most energy-dependent tissues in the body. Impaired mitochondrial metabolism may influence neuronal development and synaptic plasticity, which are crucial processes for neurodevelopment and contribute to ASD.

Meta-analyses on three case-series including 536 patients with ASD showed 5% of overall prevalence of primary MD in ASD. However, almost 30% of children with idiopathic ASD have an increase of lactic acid in plasma with respect to normal reference values [8]. In addition, markers of mitochondrial dysfunctions have been identified in peripheral tissues such as skeletal muscle [9], blood leukocytes [10,11], buccal mucosa [12], and in the brain of ASD patients by in vivo magnetic resonance spectroscopy [13]. On a clinical ground, children with idiopathic ASD may exhibit features of a MD, such as modified rate of head circumference growth, seizures, motor delay, gastrointestinal disturbances, and regression following fever or other environmental inputs [8,9].

Mitochondrial long-chain fatty acid (LCFA) oxidation (mtFAO) is an important source of energy production in mammals. LCFAs require the carnitine-mediated translocation of long-chain acyl-CoAs from cytoplasm into mitochondria where β-oxidation takes place. β-oxidation is characterized by repeated cycles of sequential shortening of the fatty acid by two carbons via four enzymatic steps with production of one acetyl-CoA during each cycle. The adult diet mostly provides LCFAs and very low amounts of medium- or short-chain fatty acids, which can also undergo β-oxidized in the mitochondria. Inborn carnitine defects or mtFAO defects induce energy deficiencies and other complex pathogenic states affecting many tissues including brain, liver, heart, and skeletal muscle [14,15,16,17].

ASD and ASD-type behavior have been described in patients with genetic defects of mtFAO [18,19]. Likewise, biomarkers of impaired mtFAO, such as depressed free carnitine levels and an increase of long-chain acyl-carnitine species, have been identified in patients with idiopathic ASD [20,21]. A full knowledge of mtFAO metabolism in the patho-physiology of ASD is still missing, however, the recognition of deficits in energy metabolism in patients with ASD may be important for better understanding the underlying biological abnormalities and, in turn, therapeutic interventions.

Nuclear peroxisome proliferator-activated receptors (PPAR) are ligand-activated receptors with distinct physiological functions and tissue distribution. Three PPAR isoforms, α, δ, and γ, have been described, which differ in their target genes, physiological functions, and tissue distribution [22,23]. PPARα and δ are preferentially involved in the control of β-oxidation in organs with high energy demands like the heart, skeletal muscle, liver, or kidneys [24,25,26,27,28], whereas PPARγ is highly expressed in peripheral tissues with high fatty acid synthesis and storage, such as adipose tissue [23,29]. Various studies document the presence of the three PPARs isoforms in neurons and glial brain cells, and support the role of these nuclear receptors in neuroprotection [29,30]. Recent evidence suggests the effectiveness of PPARα activation in amelioration of core symptoms in ASD rodent models [31]. Moreover, the PPARγ agonist pioglitazone has been used in clinical trials suggesting that PPARs might be targets for drug therapy of ASD [32,33]. Resveratrol (RSV) is polyphenolic compound, considered as a natural PPARs agonist [34,35], and is present at high levels in red grapes, nuts, pomegranates, and in the Japanese knotgrass (*Polygonum cuspidatum*). RSV has metabolic, antioxidant, and anti-inflammatory activities as well as neuroprotective effects [36,37,38,39], and its therapeutic potential has been evaluated in animal models and in humans in a wide spectrum of pathologies [40,41]. All of these biological activities may have possible applications and points of interest in ASD therapeutics, although only a few studies have been reported concerning their potential effect on ASD treatment [42,43,44,45,46].

In this study, we introduce the existing links between ASD and impaired mtFAO and review the potential implications for regulation of mtFAO in ASD by PPAR activators, including RSV.

## 2. Autism Spectrum Disorder in Genetic Diseases of Mitochondrial Fatty Acid β-Oxidation

mtFAO represents a major source of ATP in various tissues with high energy demand, in particular the heart, skeletal muscle, and liver, particularly when fasting or prolonged exercise requires glucose sparing. In these situations, adipose tissue lipolysis makes free fatty acids available for mitochondrial β-oxidation, providing acetyl-CoA that enters the tricarboxylic acid (TCA) cycle for complete oxidation. Overall, the re-oxidation of NADH and FADH_2_ produced by these processes allows large amounts of ATP production by the respiratory chain, making LCFA high-yielding energy substrates. Hepatic β-oxidation also provides acetyl-CoA for production of ketone bodies, which are used as “glucose sparing” energy substrates by the brain during fasting [16]. In addition to these bioenergetic functions, recent studies suggest that mtFAO plays a key role in providing acetyl-CoA for posttranslational modifications of cellular proteins by lysine acetylation. This process affects not only the mitochondrial compartment, in which a large number of metabolic enzymes exist in acetylated/deacetylated form [47], but also the nucleus, in which acetyl-CoA derived from mtFAO can be used as a substrate for acetylation of transcription factors and transcription co-activators, but also, importantly, of histones [48,49]. Thus, carbons stemming from fatty acids integrate into the epigenome by histone acetylation, and it is known that increases in histone acetylation are associated with chromatin relaxation, facilitated access of transcription factors, and activation of gene expression. Accordingly, this epigenetic regulation of gene expression establishes a functional link between energy metabolism, chromatin status, and gene expression [50]. How genetic and environmental heterogeneity affect specific molecular pathways in individuals with ASD is not well understood. It has been proposed that epigenetic changes driven by genetics and environmental stressors may negatively influence biological pathways important for brain development. Methylation changes have been found in several ASD candidate genes, such as the gene encoding the oxytocin receptor (OXTR) as well as RELN and SHANK3 genes. Moreover, changes in DNA methylation have been confirmed in studies aimed at directly defining ASD-specific epigenetics patterns [51]. Recently, a histone acetylome-wide association analyses was conducted on postmortem ASD brain samples and matched control brains. The study showed aberrations in histone acetylation in the prefrontal and temporal cortex of patients with ASD. It is noteworthy that the aforementioned epigenetic modifications were found to affect genes related to ion channels, synaptic function, and epilepsy/neuronal excitability, all of which have previously been shown to be dysregulated in ASD [52].

More than fifteen inborn errors of mtFAO have been described so far [16], which are caused by the deficiency of a single mitochondrial enzyme in the β-oxidation pathway. Briefly, the mtFAO defects mainly affect one of the components of the LCFA import shuttle, or one of the acyl-CoA dehydrogenase isoforms, such as short chain acyl-CoA dehydrogenase (SCAD), medium chain acyl-CoA dehydrogenase (MCAD), very long chain acyl-CoA dehydrogenase (VLCAD), or the mitochondrial trifunctional protein [long chain 3-hydroxyacyl-CoA dehydrogenase (LCHAD)] [16,17]. Mitochondrial β-oxidation deficiencies may also be secondary to defects of dedicated processes, for example, due to mutations in the carnitine transporter genes, or due to deficiencies in the mitochondrial electron transfer flavoprotein system (multiple acyl-CoA dehydrogenase deficiency) [14,16]. Molecular studies have revealed a great variety of gene mutations associated with inborn errors of mtFAO, all transmitted as autosomal recessive traits in humans. Clinically, FAO disorders present with overlapping features such as hypotonia, muscle weakness, cardiomyopathy, liver failure, encephalopathy, seizures, developmental delay/regression, and behavioral problems. Clinical presentation is highly variable, ranging from severe neonatal forms with sudden death or life-threatening multi-organ failure (Reye-like syndrome), up to mild adult-onset phenotypes [14,15].

ASD and ASD-type behavior have been reported in patients with genetic defects of mtFAO, such as VLCAD deficiency [18]. ASD is the main neuropsychiatric feature of patients with deficiency of the LCHAD enzyme, which has a high specificity for the degradation of fatty acids with 12–16 carbon chain lengths. It has been hypothesized that in VLCAD and in LCHAD deficiency, energy defects and accumulation of unmetabolized intermediates might have a particular detrimental effect on the brain during development and in adults [19,53].

Deletion of the TMLHE gene, which is part of the carnitine synthesis pathway and located on the X chromosome, is found more often in male-male multiplex families with non-dysmorphic autism, suggesting that TMLHE deficiency is a risk factor for ASD, albeit with low penetrance (estimated at 2–4%) [54]. As a whole, the occurrence of ASD-like behavior or ASD in genetic defects of carnitine biosynthesis and mitochondrial β-oxidation supports the role of dysfunctional mitochondrial β-oxidation as a possible mechanism underlying ASD, at least in a patient subset.

## 3. Fatty Acids in the Energy Metabolism of the Central Nervous System in Autism Spectrum Disorder

It is often assumed that the adult brain does not oxidize fatty acids, since neurons mainly use glucose and lactate for energy production [55,56]. However, fatty acids can enter the brain and be oxidized in astrocytes [56], and they likely represent an important oxidative fuel during embryonic and early postnatal development in rodent neural cells [56,57]. The carnitine palmitoyl transferase (CPT) system, which mediates the entry of LCFAs into the mitochondria for β-oxidation, is expressed in astrocytes [56,57], and in embryonic [58] and adult [59] neural stem cells (NSC). Recent data in mice indicate that pharmacological inhibition or conditional deletion of the CPT system alters NSC homeostasis in the embryonic and adult brain as well, suggesting that FAO might have an instructive role in neural cell differentiation [59]. In line with this, it is noteworthy that neuronal migration defects and brain dysgenesis have been described in human fetuses with the most severe presentation of inborn Carnitine Palmitoyl Transferase 2 (CPT2) deficiency [60], and in other genetic mtFAO disorders [61]. Accordingly, alterations in NSC homeostasis might account for the link between FAO defects and neurodevelopmental diseases such as ASD [57,58,59].

### Evidence of Dysfunctional Mitochondrial Fatty Acid β-Oxidation in Patients with Idiopathic Autism

In patients with idiopathic ASD [62], as well as in patients with genetic defects of mtFAO [63], developmental plateau and/or neurological regression triggered by metabolic stress (fasting), or by immune activation such as fever and infections, may follow a period of initially normal infant development. Actually, mitochondrial dysfunction may ensue during a period of metabolic stress (fasting), inducing catabolism, or as a result of immune activation, inducing inflammatory response and high rates of ATP consumption. Likewise, symptoms of mtFAO deficiencies can be provoked or aggravated by energy-requiring states such as fasting, infection, or a combination of both. For example, during starvation, reduced energy supply to the liver will ultimately result in hyperammonemia potentially responsible for brain edema, encephalopathy, and Reye-like syndrome [14,15,16,17]. Encephalopathy with high fever and severe brain damage can also be observed in influenza-infected patients harboring DNA variants of the CPT2 gene [64].

Metabolic abnormalities that occur in genetic defects of mtFAO are also seen in patients with ASD and include low plasma free carnitine levels and increase of saturated and unsaturated long-chain acyl-carnitines species, as well as hyperammonemia, abnormal plasma amino acid levels and elevated urinary excretion of TCA metabolites and dicarboxylic acids [8,20,21].

Previous clinical studies showed the existence, in subsets of patients with ASD, of elevated blood fatty acids, particularly LCFAs and very LCFAs [20,65,66]. Accumulation of LCFAs or their CoA or carnitine esters primarily inhibits energy metabolism at various steps, such as fatty acid synthesis, TCA cycle functioning, glutamate metabolism, and oxidative phosphorylation or secondarily by inducing carnitine, and coenzyme A or fatty acid binding protein depletion [67,68].

Furthermore, patients with ASD show a unique plasma acyl-carnitine profile with an increase in unmetabolized long-chain and short-chain acyl-carnitines [20,69]. We recently investigated an easily testable blood metabolic profile, including acyl-carnitines and amino acids, using high throughput analyses of samples extracted from dried blood spots (DBS). Out of 45 analyzed metabolites, nine (20%) were significantly increased in ASD patients, including the amino acid citrulline and acyl-carnitines C2, C4DC/C5OH, C10, C12, C14:2, C16, C16:1, and C18:1 (*P* < 0.001). By a naïve Bayes algorithm, we found an increased performance of the algorithm based on the identified acyl-carnitines for classifying ASD in toddlers (*n*: 42 subjects; mean age 3.26 ± 0.89) with 72.3% sensitivity (95% CI: 71.3;73.9), 72.1% specificity (95% CI: 71.2;72.9), and diagnostic odds ratio (DOR) of 11.25 (95% CI: 9.47;17.74). Considering the heterogeneity of ASD, we suggest that metabolic profiling may support the identification of phenotypes, enabling individualized therapeutic approaches in children at risk of developing the disease [70]. ESI-MS/MS analyses of different metabolites in DBS represent a high throughput method for metabolic profiling of individuals with ASD by a single injection, in a rapid, low-cost, and suitable procedure. Moreover, DBS-MS application has inherent advantages, such as collecting a small sample volume that is easily transported. However, it should be noted that several parameters can impact the accuracy of DBS measurement. In particular, robust, independent reference intervals for DBS analytes should be established [71].

The role of dysfunctional mtFAO in autism is supported by the observation that intracerebral or systemic administration of propionic acid (PPA) to adult rats induces repetitive movements, hyperactive behavior, and seizure activity, consistent with the rodent ASD model known as the PPA model [72]. In the PPA model, the induced ASD behavior is associated with the increase of total, long-chain, and short-chain acyl-carnitines in brain tissue. Thus, the rodent PPA model of ASD suggests a link between an abnormal acyl-carnitine profile in the brain and ASD behavior [73].

Physiologically, endogenous PPA acid derives from the catabolism of branched-chain amino acids and from odd-chain fatty acid catabolism. Moreover, PPA, along with short chain fatty acids such as acetate and butyrate, are major metabolic products of enteric bacteria enriched in stool samples of patients with ASD, and have been shown to modulate mitochondrial function in cell lines from patients with ASD [73,74,75]. PPA accumulation is toxic for the CNS by interfering with cellular metabolism and cell-to-cell communication and increasing inflammatory response [75]. In humans, detrimental effects of PPA accumulation are dramatically illustrated by propionic acidemia (PA). PA is a severe organic acidemia caused by a defect of propionyl-CoA carboxylase and PPA accumulation in body fluids and tissues. Patients with PA have neurological impairment and ASD or ASD behavior [76].

To summarize, the acyl-carnitines profile observed in subsets of ASD patients and in PPA rodent ASD model support the hypothesis of a partial mtFAO deficiency, but it appears relatively atypical compared to the profiles observed in inborn monogenic disorders affecting one of the enzymes in the mtFAO pathway. However, partial defects in the activity of several enzymes in the mtFAO pathway might lead to relatively unspecific acyl-carnitine accumulation. Furthermore, partial defects in mtFAO can also be related to respiratory chain (RC) dysfunction, since there are strong functional links between the mtFAO and RC pathways and increasing evidence shows that mitochondrial dysfunction associated with ASD includes deficient activity levels of both the mitochondrial RC and FAO pathways [77,78].

## 4. Molecular Regulation of Mitochondrial FAO: The PPAR Pathway

It is presently assumed that partial defects in mitochondrial functions might be a determining factor in the pathophysiology of ASD and other neuropsychiatric disorders [8,79,80]. In line with this, it can be thought that some of these disorders may be treatable by means of bioenergetic interventions [78,79,80]. The activity of mitochondrial energy producing pathways, and in particular of mtFAO, is regulated by way of changes in the transcription of genes encoding the various enzymes in these pathways. The PPARα, δ, and γ are ligand-activated receptors that bind a variety of endogenous LCFAs or their derivatives (prostaglandin, leukotrienes) [23]. Upon activation by these natural ligands, PPARs bind specific recognition sequences, called PPAR-response elements, in the promoter regions of target genes, leading to marked up-regulation of gene transcription [25,29,81]. The resulting increases in mRNA abundance translate into increased proteins and enzyme activity, and allow the coordinated up-regulation of numerous mtFAO enzymes, resulting in significant PPAR-driven increases in the mtFAO flux [24,26,29]. An additional important regulator of mtFAO is the transcription co-activator PGC1-α. This co-activator acts as a recruiting platform to bind and activate a number of transcription factors, including the PPARs, and, accordingly, mediates a variety of stimulatory effects on mitochondrial bioenergetics across multiple organs, including the brain [82,83]. The PGC1-α mediates transcriptional co-activation by phosphorylating or methylating histones, remodeling chromatin, and recruiting RNA polymerase [81]. Importantly, the expression of the PGC1-α gene can vary in response to physiological or metabolic stress conditions (i.e., exercise, starvation), is positively regulated by the PPARs, and represents a master regulator of mitochondrial functions under physiological conditions, and in a variety of neurological, metabolic, or aging diseases [82,83,84].

### 4.1. PPARs Activators for Treatment of Mitochondrial FAO Defects

PPAR receptors have attracted much attention, due to the existence of various synthetic activators, some of which are commonly prescribed drugs in human medicine. Indeed, fibrates (activators of PPAR α and beta-δ) and thiazolidinediones (TZD; PPAR γ ligands) are used for the treatment of dyslipidemia and diabetes, respectively [25,26,85]. There are also consistent data showing that genetic mtFAO defects, including CPT2 [86] and the VLCAD deficiency [87], can be corrected in fibroblasts from affected patients after treatment with bezafibrate. Thus, exposure to bezafibrate up-regulates CPT2 and VLCAD proteins and enzyme activities, and induces a coordinated increase in the proteins and enzyme activities of respiratory chain complexes I to V in human fibroblasts [15,88]. Interestingly, CPT2-, VLCAD-, or glutaric acidemia type II - deficient fibroblasts exhibit specific patterns of acyl-carnitines accumulation, which are normalized following cell treatment by bezafibrate [15,89,90]. In line with this, an open label trial in six patients with inborn defects in CPT2 showed that bezafibrate treatment stimulated long-chain FAO in the skeletal muscle, improved physical activity, and reduced the myopathic manifestations of the disease [91,92]. Bezafibrate is also presently tested in adults with a mitochondrial myopathy [93], on the basis of *ex vivo* observations showing that bezafibrate can restore inborn respiratory chain defects by stimulating the expression of deficient protein and mitochondrial biogenesis [88]. Besides inborn deficiencies of FAO, it was shown that bezafibrate upregulates CPT2 and energy metabolism in fibroblasts of patients with influenza-associated encephalopathy (IAE) [64,94]. IAE is particularly frequent is east Asians and is characterized by high fever following viral infections, severe brain damage, and multi-organ failure, with high rate of mortality and disability. IAE has been associated with a thermolabile CPT2 enzyme phenotype, and, at the molecular level, with the CPT2, F352C, and F352C+V368I gene variants. The bezafibrate-induced transcriptional upregulation of CPT2 and amelioration of cellular ATP levels in fibroblasts from patients with IAE at 37 °C and even at 41 °C suggests possible therapeutic use in patients with IAE [64].

### 4.2. PPARs and Neuropsychiatric Diseases

Various studies document the presence of the three PPAR isoforms in CNS neurons and glial cells, and support the role of these nuclear receptors in cognition and behavior [30,83]. In this regard, a transplacental influence of the maternal PPARγ variants on human fetal brain development has been demonstrated. Offspring from mothers bearing the wild allele outscore those of mother with the polymorphic Pro12Ala in cognitive, language, and motor development at the age of 18 months [95]. Several drugs of the fibrate group widely prescribed as hypolipidemic drugs, including bezafibrate, fenofibrate, gemfibrozil, and ciprofibrate, are activators of the PPARs, although they do not activate all PPAR isoforms equally [22]. Numerous studies in rodents have shown that administration of PPAR agonists up-regulated gene expression levels, resulting in greater levels of enzymes and proteins of mitochondrial oxidative metabolism, and stimulated mitochondrial biogenesis in various tissues [96,97]. Pharmacological activation of PPARs is also known to increase the expression of its own co-activator, the transcription co-activator PGC1-α (PPARγ coactivator 1-α) through a PPAR-response element in the PGC1-α promoter [98]. Accordingly, upon activation by a PPAR agonist, the PPAR-PGC1-α system forms a feedforward loop to transcriptionally amplify mitochondrial energy production while promoting mitochondrial biogenesis. Consistent with this, administration of bezafibrate was shown to increase PGC1-α expression and stimulate mitochondrial biogenesis and antioxidant defense in the striata of mice modeling Huntington’s disease. These metabolic changes were associated with improvements in motor coordination, motor deficits, and survival [97].

PPARs regulate inflammatory pathways by controlling gene expression of key transcription factors such as NF-κB or cyclooxygenase [30,99]. Activation of PPARα and PPARγ by selective agonists has been reported to exert neuro-protective effects by reducing oxidative stress and neuroinflammation, which are processes involved in ASD pathophysiology.

Since mitochondrial dysfunction is considered as a possible determining factor in the pathogenesis of neuropsychiatric diseases including ASD, some authors have explored the potential of fibrates or TZD in animal models of these disorders [30,84,99]. Most recently bezafibrate, a pan-PPAR agonist, has been tested for bipolar depression in a proof-of-concept clinical trial (NCT02481245) [83].

### 4.3. Preliminary Evidence of Natural FAO Activator Resveratrol in ASD

An increasing number of studies have been focusing on the effects of RSV in animal models of ASD. Prenatal exposure to RSV attenuates the ASD-like behavior in the model induced by prenatal exposure to valproic acid (VPA), although the molecular mechanisms mediating the effects at the cellular level are still largely undiscovered [42]. RSV lessens the effects of VPA-induced depletion of GABAergic parvalbumin (PV+) neurons in sensory brain regions and in the amygdala [44]. Recent evidence shows that RSV exerts epigenetic effects which counteract the effects of environmental factors such as VPA on molecular targets of ASD. MicroRNAs (miRNAs) are a group of small noncoding RNA molecules which can regulate gene expression at the post-transcriptional level. Dysregulation of miRNA may lead to abnormal DNA methylation and a change in the expression of ASD-related genes. The study of miRNA has added information on ASD pathogenesis and might become helpful to better discriminate between different disease subgroups. In this regard, we recently validated serum miR-140-3p as significantly upregulated in ASD patients compared to healthy controls and we found that miR-140-3p may discriminate against patients with a unique ASD diagnosis from those with ASD associated to a comorbid disorder (ASD and Tourette syndrome) [100]. Interestingly, RSV-dependent amelioration of social behavior in a VPA model runs parallel to a decrease of miRNA miR134-5p and miR138-5p that are also upregulated in individuals with ASD [45].

The putative mechanisms of RSV effectiveness in models include regulation of abnormal neuroimmune response in ASD. RSV-treated BTBR mice, a mouse model largely validated for ASD, had significant decrease in IL-6, TNF-α, IFN-γ, and STAT3 in CD4+ spleen cells when compared to BTBR control mice. RSV treatment also decreased IL-6, TNF-α, IFN-γ, JAK1, and STAT3 mRNA expression levels in the brain tissue when compared to BTBR control mice [101].

Interestingly, RSV reversed the effects of prenatal progestin-exposure-induced ASD-like behavior through the activation of Estrogen Receptor β (ERβ) and its target genes in the amygdala. As a consequence, RSV significantly improved ASD-like behavior in the model. Behavioral amelioration was associated with improved mitochondrial function and FAO flux, demonstrated by measuring palmitate oxidation rate in vivo [46]. In line with evidence in experimental models of ASD, the effects of RSV in fueling mtFAO have been demonstrated in cultured human fibroblasts of patients with genetic FAO defects. RSV enhances residual CPT2 activity in fibroblasts harboring CPT2 gene mutations and restores normal FAO rates [102]. Similar effects were also documented in fibroblasts from patients with VLCAD deficiency, suggesting that RSV can improve mtFAO in human FAO-deficient cells [103].

Currently, the molecular mechanisms of RSV-induced FAO regulation are not completely understood. It has been shown that RSV increases the activity and gene expression of sirtuin 1 (SIRT1), associated with an increase in CPT1 mRNA, encoding the rate-limiting enzyme of mtFAO. Moreover, SIRT1 activation could result in PGC-1α activation, and in the transcriptional co-activation of nuclear and mitochondrial genes encoding for proteins involved in mitochondrial biogenesis, oxidative phosphorylation, and energy production. Finally, post-translational SIRT1 activation by AMP-activated protein kinase could further enhance FAO and mitochondrial biogenesis [103,104].

### 4.4. PPARα and Autism Spectrum Disorder

The implication of PPARs in lipid homeostasis and the greater risk for obesity and dyslipidemia in ASD compared to the general population [105], suggest a role for PPARα in metabolic dysregulation in patients with ASD. PPARα expression and function have been explored in various preclinical models of ASD. PPARα expression is depressed in the frontal cortex of the VPA rodent model of ASD (VPA-model) [106], whereas the administration of palmitoylethanolamide (PEA), an endogenous lipid agonist of PPARα, reduced autistic behavior in the VPA-model of ASD [107]. The effects of PPARα activation were analyzed in a BTBR T+tf/J (BTBR) mouse model. PPARα activation associated with selective PPARα agonists [31] and with PEA supplementation [108] reduced the repetitive behavior and stereotypes in the BTBR mouse model.

PPARα deficient mice have a distinct cognitive and behavioral phenotype characterized by reduced spatial information processing and cognitive flexibility, along with repetitive/perseverative behavior reminiscent of that seen in preclinical models of ASD. Morphological and functional analysis in PPARα deficient mice showed an impairment of parvalbumin-positive GABA-ergic interneurons in the frontal cortex and in the hippocampus, resulting in cortical excitation [31]. As expected, PEA administration failed in inducing its effect to ameliorate stereotyped behaviors in PPARα knockout mice [108]. Based on this evidence, PEA was used as an adjunctive therapy to treat irritability and hyperactivity symptoms in children with ASD receiving risperidone, with significant effects compared to risperidone plus placebo [109]. These findings collectively link PPARα activation with improved behavioral phenotype in ASD preclinical models, and support the role of PPARα as therapeutic target for drug development in ASD.

### 4.5. PPARγ and Autism Spectrum Disorders

TZD are PPARγ agonists that act as anti-diabetic drugs due to insulin-sensitizing effects. TZD molecules share anti-inflammatory properties and have been used in a variety of inflammatory diseases such as atherosclerosis, psoriasis, and inflammatory bowel disease [29]. Pioglitazone is a TZD approved and commercialized for treatment of type I diabetes, also in adolescence [110]. Pioglitazone reduces inflammatory glia activation and improves glucose utilization and lactate production in brain glial cells [111]. The use of pioglitazone in ASD patients has been considered, based on evidence that links ASD to immune dysregulation [109], occurrence of autoimmune diseases in children with ASD [110], and neuroinflammation [112]. In particular, altered immune response in patients with ASD include abnormal T lymphocyte function, increased cytokines of Th2 and Th1 arms of the immune response, and neuroinflammatory changes seen in autoptic brains consistent with microglia and astroglia activation [113,114]. Evidence of the intrinsic relation between neuroinflammation and ASD have been extensively reviewed [115].

Based on neuroprotective and anti-inflammatory properties, in 2007 pioglitazone was first used in a clinical trial including twenty-five children and adolescents with age ranging from 3 to 17 years. Participants showed high prevalence of auto-immune comorbidities and allergic diseases. In this cohort of patients with ASD, pioglitazone treatment at the dosage 30–60 mg per day for 3–4 months was effective in amelioration of social behavior by reducing irritability, lethargy, stereotypy and hyperactivity measured using the Aberrant Behavior Checklist, particularly in younger patients. No adverse effects were recorded in the study period [32]. Later on, pioglitazone was studied as a treatment adjunct to risperidone in children with ASD aged 4–12 years. A 10-week randomized, double-blind, placebo-controlled trial supported the safety profile and its role in controlling behavioral symptoms of ASD [33]. The efficacy of pioglitazione in ASD was related to different effects of PPARγ activation, such as reduction of brain glia-mediated inflammatory response, improvement of brain mitochondrial function, and activation of the met proto-oncogene (hepatocyte growth factor receptor) (MET) signal transduction pathway [116]. Although safety profiles of TZD were documented in young patients with type 1 diabetes [110], further studies are required to understand the effects on behavior and long-term safety of TZD in children with ASD. Novel approaches using natural FAO activators like RSV are particularly appealing, due to their proven application for correction of CPT2- and VLCAD-deficiency in patient cells [103,117].

## 5. Concluding Remarks

There are currently no US Food and Drug Administration-approved drugs for the core symptoms of ASD, although several agents demonstrate promise for the treatment of social deficit symptoms [118]. ASD is a highly heterogeneous condition with variable underlying mechanisms. Recognition of specific classes of ASD patients by biological markers has been considering effective for better understanding molecular mechanisms and to guide tailored therapeutic strategies. If the exact nature of mitochondrial dysfunction(s) in ASD patients, and its tissue distribution, remain unclear as yet, it can nevertheless be suggested that pharmacotherapy targeting the FAO or RC might be quite relevant to test.

During the last decade, a large number of studies have been dedicated to the characterization of various biological properties of RSV, and it is now known that improvement of mitochondrial functions is a key element that accounts for RSV’s beneficial effects. These observations suggest possible helpful applications of RSV in many diseases such as cancer, cardiovascular, metabolic (diabetes, obesity), or neurodegenerative disorders (Alzheimer and Parkinson) [41]. Valuable in vivo data have been gathered, supporting a good safety profile and tolerability of RSV in humans [119].

**Figure 1:**
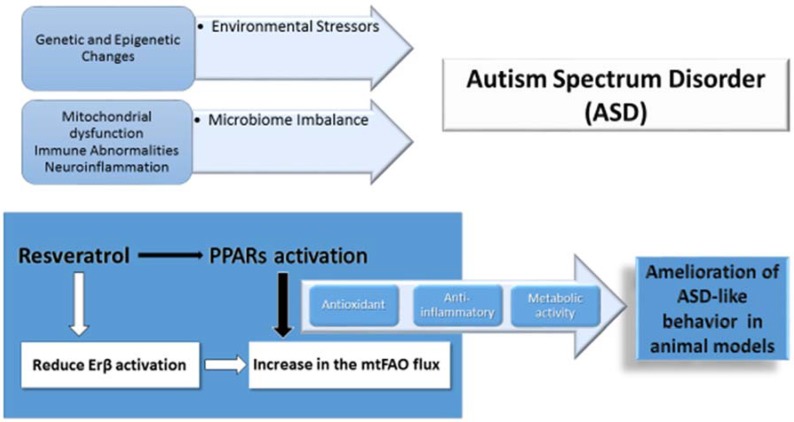
Pathogenetic mechanisms of autism spectrum disorder (ASD) and related resveratrol (RSV) actions with potential therapeutic effects proven in ASD animal models.

Considering all available data, we suggest an additional biological involvement of RSV in the treatment of ASD via regulation of mtFAO and energy homeostasis (Figure 1). Future studies in this domain might be directed toward (1) testing the occurrence of allelic variations responsible for heat inactivation of CPT2 and other pivotal components of ATP generation through mtFAO. This topic is particularly important in children with regressive ASD apparently related to infections and high-fever episodes; (2) ex-vivo approaches in patient cells might be helpful to understand the effectiveness of RSV and synthetic PPAR activators in improving energy metabolism in ASD; (3) further studies using ASD models including a PPA rodent model could help to clarify whether activation of metabolic FAO using RSV and correction of related metabolic changes, such as abnormal acyl-carnitine profiles, run parallel to behavioral amelioration. Naturally occurring activators of FAO like RSV and synthetic PPARs agonists with proven activity in regulating FAO currently used in clinical practice might thus become available for clinical trials in patients with ASD.

## Abbreviations

ASD	autism spectrum disorder
CPT	carnitine palmitoyl transferase
CNS	central nervous system
ERβ	Estrogen Receptor β
ESI-MS/MS	Electrospray ionization tandem mass spectrometry
FAO	fatty acid oxidation
IAE	influenza associated encephalopathy
IEM	inborn errors of metabolism
LCFA	long-chain fatty acid
LCHAD	long chain 3-hydroxyacyl-CoA dehydrogenase
MCAD	medium chain acyl-CoA dehydrogenase
MET	met proto-oncogene
miRNA	microRNA
mtFAO	mitochondrial long-chain fatty acid oxidation
NSC	neural stem cells
PPAR	peroxisome proliferator-activated receptors
PGC-1α	PPAR γ coactivator 1-α
PA	propionic acidemia
PEA	palmitoylethanolamide
PPA	propionic acid
RC	respiratory chain
RSV	resveratrol
SCAD	short chain Acyl-CoA dehydrogenase
SIRT1	sirtuin 1
TCA	tricarboxylic acid
TZD	thiazolidinediones
VLCAD	very long chain acyl-CoA dehydrogenase
